# Supplementary material for: Active Low-Density Polyethylene-Based Films by Incorporating α-Tocopherol in the Free State and Loaded in PLA Nanoparticles: A Comparative Study
Source: Foods. 2024 Feb 2;13(3):475. doi: 10.3390/foods13030475 (PMC10855272; doi:10.3390/foods13030475)
Supplement: Supplementary file 1 [file foods-13-00475-s001.zip › foods-2775088-supplementary.pdf]

Supplementary Materials:

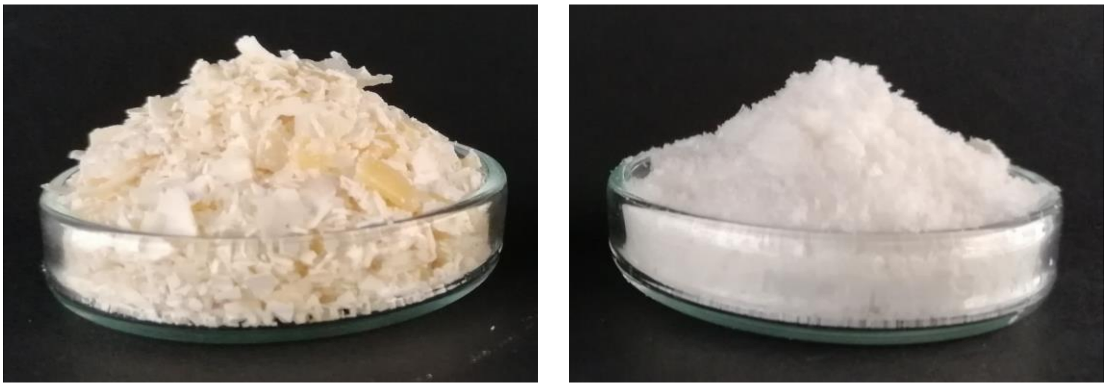

(**a**) (**b**)

Figure S1: Image of PLA NPs loaded with α-TOC dried by: A) oven and B) freeze drying.
